# Ultrafast time-resolved small-angle X-ray scattering setup on the FemtoMAX beamline

**DOI:** 10.1107/S1600577526006508

**Published:** 2026-07-28

**Authors:** J. C. Ekström, P. Mota-Santiago, F. Engelmann, M. Leorato, B. Ahn, D. Kroon, J. Larsson, A. E. Terry, A. Jurgilaitis

**Affiliations:** a MAX IV Laboratory Lund University PO Box 118 SE-221 00Lund Sweden; b Australian Synchrotron, ANSTO, Clayton, Victoria3168, Australia; c Institute for Cybersecurity and Digital Trust The Ohio State University Columbus Ohio USA; d European Spallation Source ERIC Box 176 SE-221 00Lund Sweden; e Department of Physics Lund University PO Box 118 SE-221 00Lund Sweden; f LINXS Institute for Advanced Neutron and X-ray Science, Scheelevägen 19, SE-22370Lund, Sweden; European XFEL, Germany

**Keywords:** time-resolved small-angle X-ray scattering, ultrafast X-ray scattering, ultrafast processes, laser pump X-ray probe, Au nanoparticles

## Abstract

The design and main characteristics of the ultrafast time-resolved small-angle X-ray scattering (TR-SAXS) setup on the FemtoMAX beamline at MAX IV are described. TR-SAXS experiments have successfully been performed in a transmission geometry investigating solid gold nanospheres sealed in a quartz capillary. The femtosecond laser pump and X-ray probe technique has been used to commission the setup and follow the dynamics in the laser-excited solid nanoparticles. This commissioning experiment on the FemtoMAX beamline has revealed picosecond thermal expansion dynamics in gold nanostructures. The results demonstrate the beamline’s potential for probing ultrafast phenomena in condensed matter systems employing the SAXS technique. This method complements the available diffraction techniques on the FemtoMAX beamline and opens new capabilities for investigating light-induced structural dynamics in solid-state materials.

## Abstract 2

The feasibility of performing time-resolved small-angle X-ray scattering measurements with picosecond temporal resolution on the FemtoMAX beamline at the MAX IV Laboratory in Sweden is demonstrated.

## 1. Introduction

Small-angle X-ray scattering (SAXS) is a powerful method that can be used to characterize the structure of solid materials on the nanometre scale. Detailed method descriptions have been given by Glatter (1977 ▸) and Pilz *et al.* (1979 ▸). The SAXS method is broadly applied for investigating different classes of samples from solid nanoparticles (Simmler *et al.*, 2023 ▸; Plech *et al.*, 2006 ▸) to thin films (Yokoyama, 2013 ▸) and magnetic nanostructures (Honecker *et al.*, 2022 ▸). SAXS is also applied in life science applications where proteins and large biological macromolecules are studied (Blanchet *et al.*, 2023 ▸; Kikhney & Svergun, 2015 ▸).

The size and shape of a scattering entity is the main information being extracted from SAXS measurements. It is known that the size and shape of a nanostructure influence the performance of new electronic devices (Rainò *et al.*, 2018 ▸; Talapin *et al.*, 2010 ▸). SAXS can also provide information about phase separation in alloys (Okuda *et al.*, 2000 ▸). Time-resolved small-angle X-ray scattering (TR-SAXS) is a method used to follow changes in size and shape that are initiated by an external trigger which can be light (Wulff *et al.*, 2003 ▸), temperature (Rimmerman *et al.*, 2018 ▸), pH (Monsen *et al.*, 2025 ▸) or electric field (Feng *et al.*, 2019 ▸). To allow access to ultrafast timescales, SAXS can be combined with femtosecond pulse laser excitation and use of the pump–probe method (Huang, 2023 ▸). The time resolution in such experiments is limited by the duration of the pump and probe pulses, and by the timing stability or accuracy of the available timing diagnostic tools (Kroon *et al.*, 2025 ▸). Ultrafast TR-SAXS data provide average information on the sample dynamics on ultrafast time scales where the shape and size transformations can be recorded at different time positions after the initial event. These specific experiments can only be performed on short-pulse facilities such as FemtoMAX at the MAX IV Laboratory or on free-electron laser (FEL) beamlines such as Bernina in Switzerland (Ingold *et al.*, 2019 ▸). The commissioning experiment described here is focused on demonstrating the SAXS capabilities by measuring the thermal expansion in gold nanoparticles following laser excitation using a femtosecond X-ray probe pulse.

The TR-SAXS setup on FemtoMAX can be used as a complement to crystallographic measurements, where a time-resolved diffraction signal is recorded first at large scattering angles, followed by subsequent SAXS measurements. This capability allows for recording laser-induced acoustic strain propagation and comparing it with average size variations on ultrafast time scales (Jurgilaitis *et al.*, 2014 ▸).

## 2. Beamline setup

The FemtoMAX beamline is a LINAC-based laser-pump and femtosecond X-ray probe beamline operating at 10 Hz and it is dedicated to ultrafast dynamics in solids. The beamline is located at the MAX IV Laboratory in Sweden. FemtoMAX is equipped with a 666-period in-vacuum undulator with a 10 m active length that produces X-rays in a broad wavelength range (Enquist *et al.*, 2018 ▸).

For this experiment, a multilayer mirror monochromator with a bandwidth of 1.6% was set to operate at 11 keV X-ray energy. This energy was set up by tuning undulator gaps to extract the third harmonic of the undulator spectrum, which provides 720000 photons per pulse at the sample position. The X-ray flux was measured at the sample position using an AXUV100 Si diode. A toroidal mirror demagnifies the X-ray source by factor of 2.5 in the undulator to a 100 µm × 100 µm X-ray focal spot size at the sample position. The angular separation between the incident X-ray beam and the laser beam was 5°. The short laser pulses were generated by a passively mode locked titanium-doped sapphire oscillator followed by Ti:Al_2_O_3_ two-stage regenerative multipass laser amplifier operated at 10 Hz, with an average power pulse energy of 6 mJ at the sample position. The laser wavelength was centred around a wavelength λ of 800 nm and the pulse duration was measured and found to be 80 fs. To excite the gold nanoparticles sealed in a quartz capillary, laser pulses at the second harmonic (λ = 400 nm) with a peak fluence of 5 mJ cm^−2^ were used. The LINAC photocathode gun laser, the accelerating RF field and the FemtoMAX pump laser are all synchronized to a common distributed 3 GHz master clock. The RF signal is amplified in the LINAC klystrons to accelerate the electron bunch, while the lasers are synchronized by actively stabilizing their oscillator cavity lengths such that the 39th harmonic of their repetition rate is phase locked to the clock (Enquist *et al.*, 2018 ▸). The resulting pulse-to-pulse arrival time between the laser pump and X-ray probe pulse is measured using the beamline arrival-time monitor (Kroon *et al.*, 2025 ▸), which was approximately 200 fs (FWHM). X-ray scattered signal was recorded using an Andor Balor detector with a pixel size of 12 µm and 49.5 mm × 49.5 mm active area located 2.43 m from the sample position. Measurements were performed in a high-vacuum environment with a base pressure of 5 × 10^−6^ mbar.

## 3. SAXS configuration

The sample-to-detector distance for TR-SAXS on the FemtoMAX beamline can reach a maximum distance of 6 m. Several two-dimensional large area detectors with different pixel sizes can be configured to achieve different *Q* magnitudes. The beamline is equipped with three different detectors from two different vendors: Andor Balor, Andor Zyla and Dectris Pilatus 1.2M. The available maximum magnitude of the scattering vector *Q*, for the Balor detector, is summarized in Table 1 ▸. The magnitude of the scattering vector is defined as *Q* = 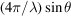, where 2θ is the scattering angle registered by a detector and λ is the wavelength of the X-rays. The angle at which the radiation is scattered is related to the size of the sample. Larger structures will cause a smaller angle of X-ray deflection and vice versa. This information is in reciprocal space, as the units of *Q* are 1/Å. The detector used in the commissioning experiment was mounted on a robot arm with six degrees of motional freedom, enabling precise manipulation and translation. The initial sample-to-detector distance of 0.1 m was calibrated using lanthanum hexaboride (LaB_6_) powder of 99% purity and 45 µm particle size, purchased from Goodfellow Advanced Materials. The value of 0.1 m is the minimum distance achievable in the setup and allows the collection of multiple LaB_6_ diffraction rings, which are then used to align the detector to normal incidence with respect to the incoming X-ray beam. The direct-beam position on the detector corner was recorded using X-ray attenuators. The detector was then translated linearly to the measurement position while maintaining a constant direct-beam position on the active area. At longer distances, LaB_6_ calibration is not feasible, therefore the detector spatial position was tracked using laser pointers in both the longitudinal and transverse directions. The estimated positional error is below 2%, primarily limited by the laser beam size and mechanical stability and by the direct measurement uncertainty.

## 4. Experimental setup

Gold nanospheres were loaded into a quartz capillary tube with an inner diameter of 1 mm and a single-wall thickness of 20 µm. The particles self-organized into a foam-like porous structure with a semi-transparent optical characteristic as a result of space charge and electrostatic interactions. The nanoparticles precipitated onto the capillary wall, leaving the centre part of the capillary empty. The capillary was sealed and placed inside the FemtoMAX grazing-incidence X-ray scattering (GIXS) endstation which is operated at 5 × 10^−6^ mbar. The detector was shielded by a 40 µm thick Al foil in order to protect it from scattered laser radiation. All factors contributing to the reduction in X-ray intensity compared with the incoming radiation are listed in Table 2 ▸.

**Figure 1:**
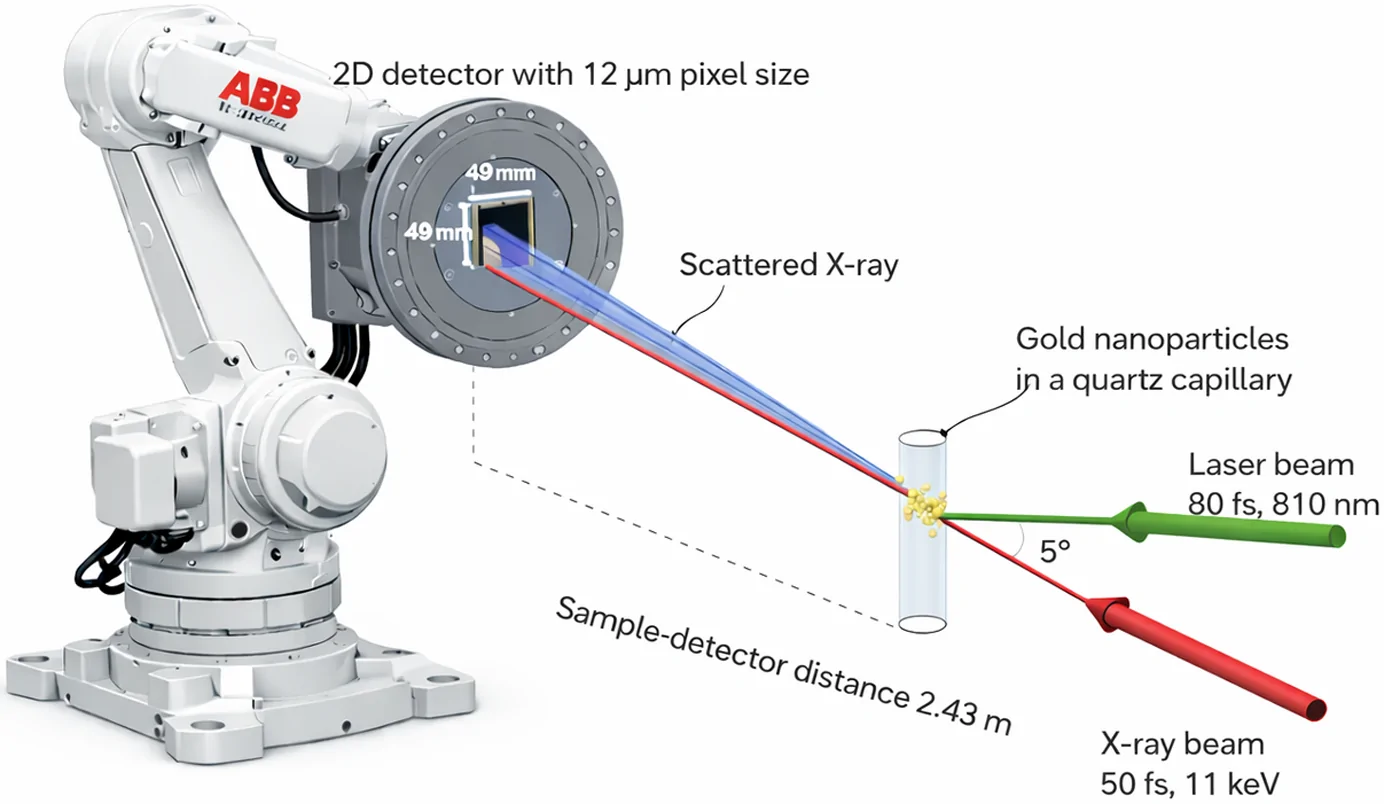
A graphical representation of the TR-SAXS setup on the FemtoMAX beamline.

The thickness of the probed gold sample layer was calculated by measuring and comparing the X-ray transmission signals from the empty quartz capillary and that loaded with Au nanoparticles using an AXUV-100 photodiode. An X-ray transmission of 95% was recorded, which corresponds to a homogenous Au layer thickness of 300 nm (Henke *et al.*, 1993 ▸). High atomic number (*Z*) materials such as gold scatter X-rays efficiently, thus they are very suitable materials to benchmark a TR-SAXS setup. The penetration depth of the 400 nm excitation laser pulse is just under 10 nm (Johnson & Christy, 1972 ▸) in solid gold; the foam-like porous structure of the gold nanoparticles guarantees a nearly homogeneous sample excitation. Uniform sample excitation was verified by measuring the transmitted laser light through the sample-filled capillary using an L50(150)A-BB-35 Ophir thermal sensor. A transmission of approximately 60% was measured, concluding that the laser fluence is reasonably uniform throughout the sample volume. We also note that the measured laser transmission fits well with the previously published findings of Loebish (1972 ▸). The experimental setup is presented in Fig. 1 ▸.

## 5. Single photon extraction from sCMOS images

**Figure 2:**
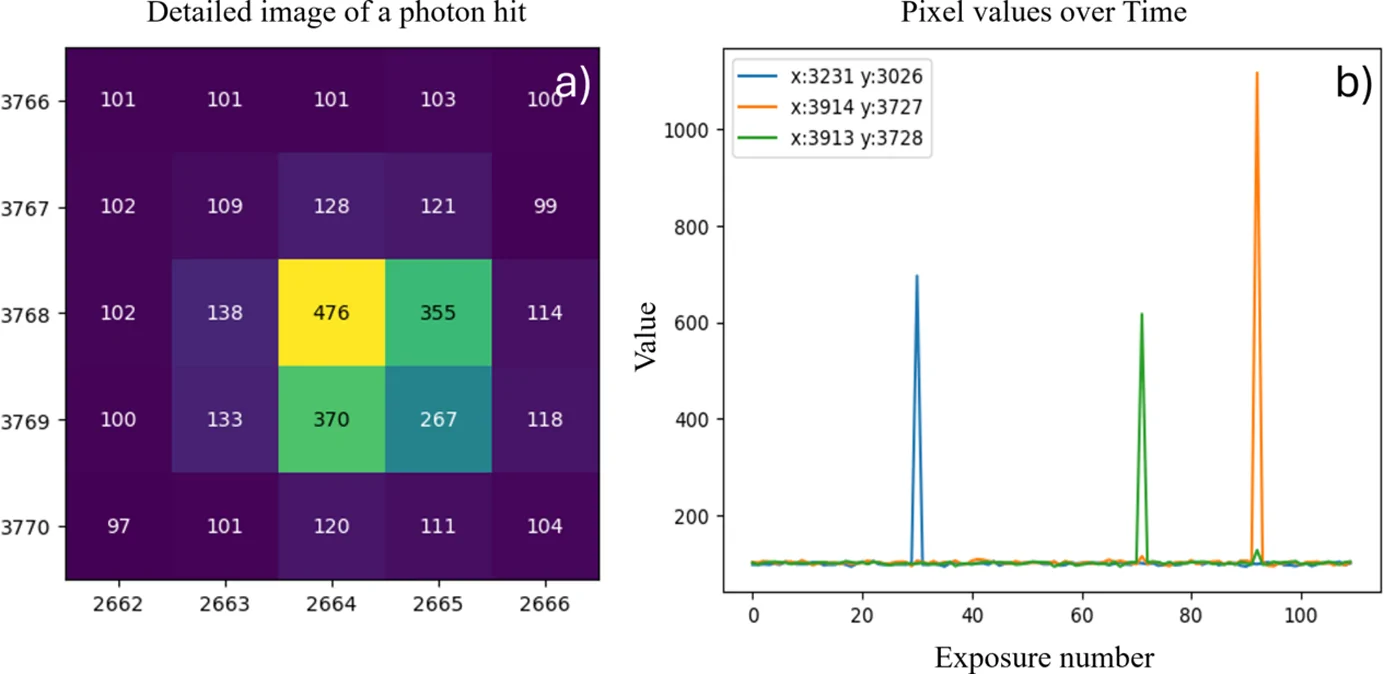
(*a*) A fraction of the raw sCMOS detetctor area, showing pixel values of a single photon hit. The *x* and *y* coordinates are the sensor position. This plot shows the main position at (2664, 3768) with charge sharing to the adjacent pixels. (*b*) Raw sCMOS values of three pixels over many exposures, where each exposure is made for a single X-ray pulse. The photon appears to have hit the *x*:3914 *y*:3727 pixel (orange trace) quite centrally as it has shared little charge with its neighbours. The other two pixels have lower peaks as more charge was shared with neighbours. Charge sharing is visible as the small peak (green) of the *x*:3913 *y*:3728 pixel in exposure/frame 92. Note that the green and orange traces are diagonal neighbours.

In this section, we describe the data processing to extract single-photon hits from the Andor Balor sCMOS camera. This is possible as the detector only captures approximately 100 photons on a 16.9 megapixel sensor. Statistically, a photon of energy *E* (in electronvolts) will yield *E*/3.6 electrons. The electrons can be shared between pixels due to hits between pixels, a particle track at an angle that intersects with multiple pixels or diffusion. This charge sharing can be significant due to the small size (12 µm) of the pixels and must be taken into account. This means that an 11 keV X-ray photon typically produces a charge distribution over several pixels, as shown in Fig. 2 ▸(*a*). The pixel intensity value above the background value is proportional to the number of electrons, but the exact factor depends on many technical factors such as the A/D (analogue-to-digital) converter settings. In our experiment, an 11 keV phonon generates about 3000 electrons, which in total gives a pixel intensity value of about 1200. In Fig. 2 ▸(*a*), most charges are distributed over nine pixels (a 3 × 3 pixel area), and we also note that the background pixel intensity is approximately 100. This background is higher than for many CCD detectors and needs special attention for sCMOS detectors.

This information from Fig. 2 ▸(*a*) was used to design the evaluation algorithms. To obtain the dark image of the detector in the current setup, we exploited the sparsity and used the median value of all pixels as a dark level. Fig. 2 ▸(*b*) shows the value of three pixels over the time of 110 exposures. The photon hits are clearly visible and the median provides a good dark value. To avoid the background and noise adding up over time, we processed every frame.

Initially we investigated the frames for values above a certain threshold. Each of the pixel positions with intensity above the threshold is a potential elastic scattering event. However, the peak intensity does not give sufficient information about the number of electrons generated by the hit, as we do not know how the deposited charges were shared between pixels. We defined a 3 × 3 (or alternatively a 5 × 5) pixel area surrounding the centre. This is sufficiently large to integrate all charges if it is positioned correctly. We then optimized the position of the pixel area to obtain the maximum value when the intensity above the background is added for each pixel within the area. The centre of gravity (COG) of the pixel area gives the position on the detector, and the sum of the intensities above the background is proportional to the number of electrons generated in the hit and is thus a measure of the photon energy.

**Figure 3:**
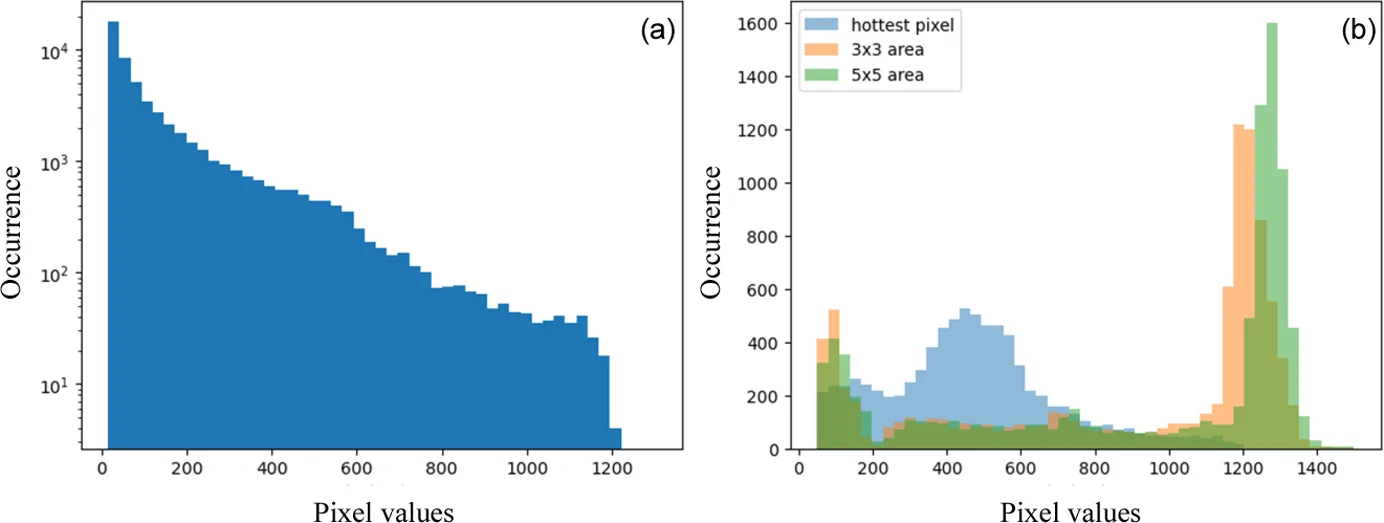
(*a*) A histogram of raw pixel values in a representative frame. It shows an exponential distribution with the hottest pixel value of 1200. Importantly, all pixel values below the maximum are present. (*b*) A histogram of the hottest pixels with a peak at 500, as most hits share more than half of the intensity with their neighbours and the central pixel only gets 500 out of 1200. Including the surrounding pixels in a 3 × 3 area or 5 × 5 area, we get a sharp peak around 1200, which shows that every photon creates the same intensity, just spread over multiple adjacent pixels.

The procedure was repeated for each frame and in Fig. 3 ▸ we show the statistics. In Fig. 3 ▸(*a*), the distribution of raw pixel values after background subtraction can be seen. This gives a nearly continuous distribution ranging from the threshold to the hottest possible value (brightest pixel value). In Fig. 3 ▸(*b*), we have identified each detected photon and plotted the hottest pixel value, the sum of the intensity of a 3 × 3 (and a 5 × 5) pixel matrix centred at the COG of the event.

**Figure 4:**
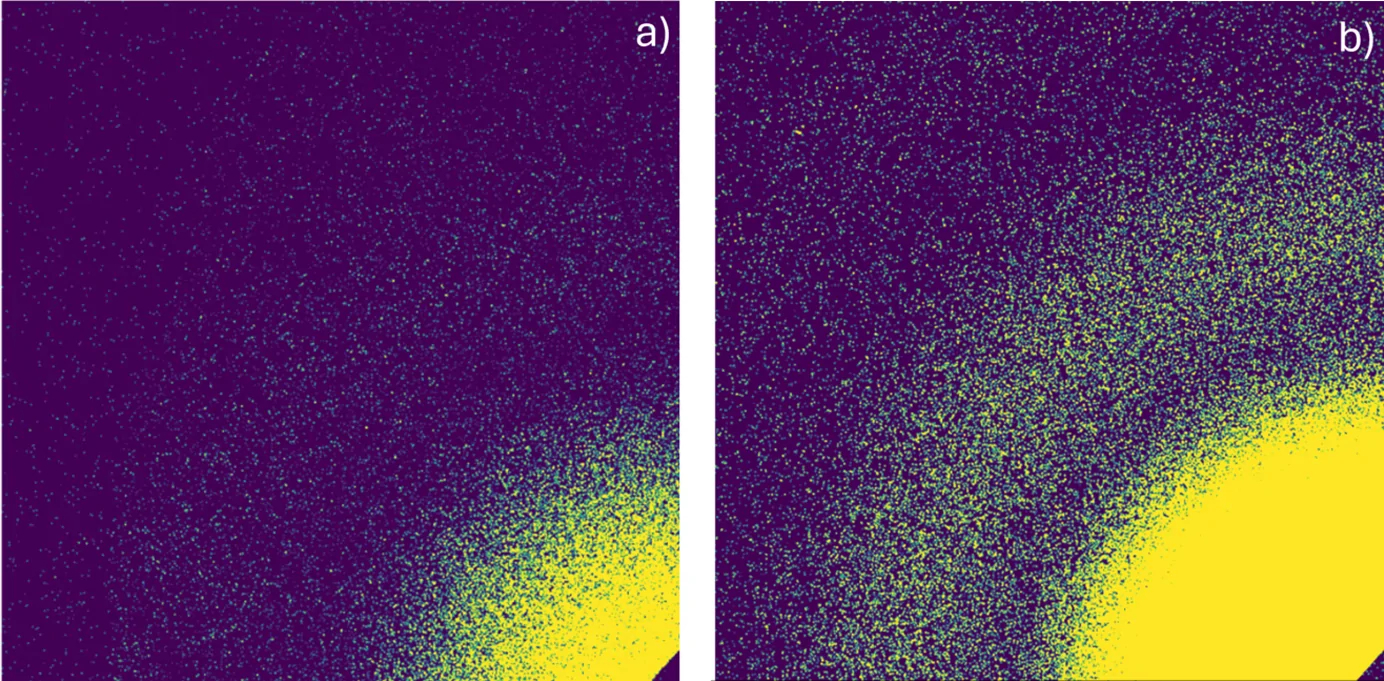
Andor Balor images. (*a*) A sum of 1000 images resulting in 2 × 10^7^ detected X-ray photons and (*b*) a sum of 5000 images resulting in 1 × 10^8^ detected X-ray photons.

The hottest pixels on their own have a peak of around 500. This clearly shows that most hits have charge sharing and we need to use the accumulated intensity with neighbouring pixels. The sum of the 3 × 3 area gives a sharp peak at 1200 and the 5 × 5 pixel area shifts the peak to about 1300. There is a statistical spread of the number of electrons generated in each hit, even if the photon energy is constant. The scientific background to the statistical spread is discussed by Owens *et al.* (2002 ▸) and references therein. Given the statistical spread, the 3 × 3 matrix gives an adequate value at a lower computational cost. We define an elastically scattered photon as one with a sum pixel area intensity higher than 1100 and use these to create 2D histograms where the photons are counted at each detector position. These are noise-free images that can be seen in Figs. 4 ▸(*a*) and 4 ▸(*b*). We also note that, by analysing the pixel value histograms such as the one in Fig. 3 ▸(*b*), one can find pile-up effects where multiple photons hit close to each other. As can be seen in Fig. 3 ▸(*b*), this did not occur under these experimental conditions since that would have resulted in a pixel value (up to a factor of two) higher than the maximum for a single photon.

The complete data analysis workflow is summarized in Table 3 ▸.

## 6. Benchmarking the ultrafast SAXS setup

First, a static scattering pattern from the solid nanoparticles was recorded. Typical static SAXS scattering patterns are shown in Fig. 4 ▸. It is possible to compare the sum scattering signals containing 1000 and 5000 X-ray pulses in Fig. 4 ▸(*a*) and Fig. 4 ▸(*b*), respectively. Gold scatters X-rays efficiently, resulting in a noticeable scattering pattern after 1000 images which corresponds to a total deposited photon flux of 2 × 10^7^ on the 16.9 Mpixel detector. The detector is offset such that the direct beam hits the bottom right-hand corner of the detector in order to reach larger *Q* magnitudes. Most of the scattered signal is deposited on the first half of the sensor and higher-*Q* regions were less populated due to less intense sample scattering at higher *Q* magnitudes. In the first 1000 shots the first SAXS scattering ring is evident, while the second one becomes noticeable after collecting 5000 X-ray pulses.

The small pixel size (12 µm × 12 µm) detector from Andor, together with the photon counting algorithm, gives an excellent spatial resolution on the detector that translates into a *Q* resolution which is more than sufficient to resolve the broad scattering rings from the sample, as seen in Fig. 4 ▸(*b*). The achieved resolution will allow us to bring the detector closer in future experiments. This will improve the signal-to-noise (S/N) ratio since more of the scattering signal will be captured by the active area of the detector if the direct beam is centred on it. The signal from the Au nanoparticles was sufficiently strong for this experiment, so the detector distance and sample positions were kept unchanged. At a repetition rate of 10 Hz, we collected 5000 X-ray shots over 8.3 min, resulting in a noise level below 2% in the 0.04 < *Q* < 0.1 Å^−1^ range. A better S/N ratio at *Q* > 0.1 Å^−1^ can be achieved by increasing the data acquisition time, changing the sample-to-detector distance and matching the X-ray energy to the maximum quantum efficiency of the detector.

To benchmark the SAXS data from FemtoMAX, static SAXS patterns were collected from both FemtoMAX and the dedicated SAXS beamline CoSAXS on the 3 GeV ring of the MAX IV Laboratory (Berntsson *et al.*, 2022 ▸). When the X-ray beam on CoSAXS is focused on the detector, the beam size is comparable with the beam size on FemtoMAX at the time of the experiment (120 µm × 120 µm FWHM). On CoSAXS, two detectors with an exposure time of 0.1 s were used to record the SAXS pattern. The first detector was a Dectris Eiger2 4M detector for SAXS and the second a Dectris Pilatus 2M detector for wide-angle X-ray scattering. The available photon flux on CoSAXS is 10 × 10^13^ photons per second at 12 keV, which results in 10 × 10^12^ photons deposited on the detector to record static SAXS patterns.

**Figure 5:**
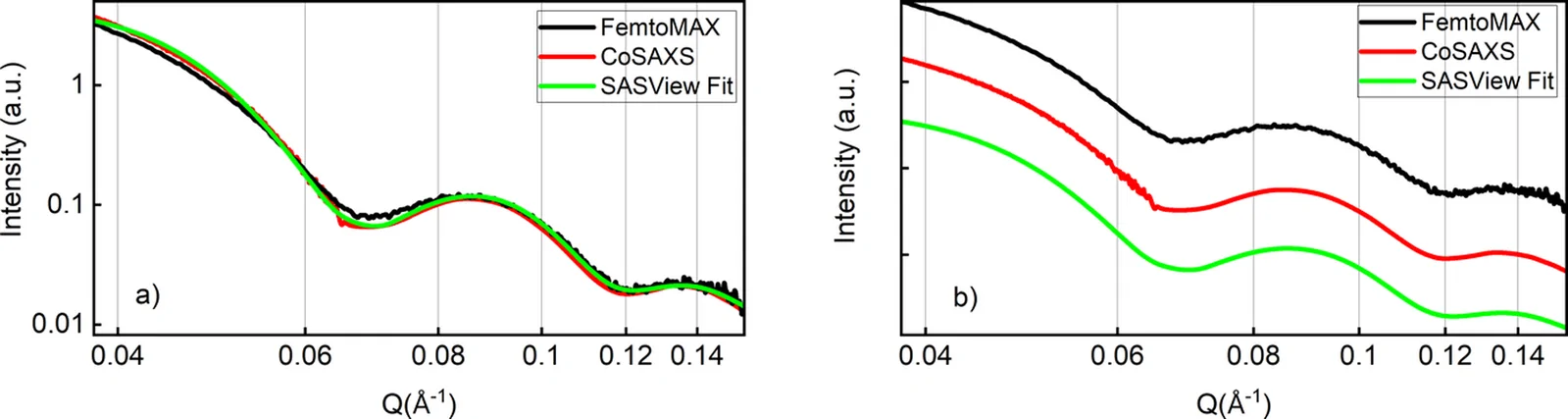
(*a*) SAXS patterns from gold nanospheres measured on the FemtoMAX and CoSAXS beamlines at the MAX IV Laboratory. The green curve is a fit to the CoSAXS data using *SasVIEW*. (*b*) The curves have been deliberately vertically offset for clarity. The FemtoMAX data have a noise level of 1.8% in the 0.04 < *Q* < 0.11 Å^−1^ range, compared with less than 1% for the CoSAXS beamline setup in the same range.

Azimuthal integrations of the two-dimensional SAXS pattern from the two different beamlines are presented in Fig. 5 ▸, together with the fit to a spherical form factor. The scattering profile from CoSAXS yields a better S/N ratio at larger *Q* magnitudes (*Q* > 0.1 Å^−1^). The larger *Q* range on CoSAXS is achieved by using and combining data from the two different detectors. The better S/N ratio on the CoSAXS beamline derives from the higher number of photons incident on the sample and the higher quantum efficiency of the Dectris Eiger detector compared with the Andor Balor detector on FemtoMAX. The *Q* resolution of the data from CoSAXS is optimized by minimizing the divergence of the incoming X-ray beam using the X-ray optics to focus the X-ray beam onto the detector plane. Other than the difference in S/N ratio and *Q* range previously mentioned, the data from the two beamlines are similar. The difference in S/N ratio is most pronounced at high *Q* > 0.1 Å^−1^ values, where Poisson­ian statistics (Saleh & Teich, 1991 ▸) govern the fundamental limit for the S/N ratio due to the comparatively lower photon flux on FemtoMAX.

The size distribution of the nanospheres can be extracted from the integrated SAXS patterns. There are many software packages available for the analysis of SAXS patterns, such as *SASfit* (Breßler *et al.*, 2015 ▸), *Irena* (Ilavsky & Jemian, 2009 ▸) and *SasVIEW* (https://www.sasview.org/). In the present study, we used *SasVIEW* to extract sample information from the SAXS pattern by fitting the data with a polydisperse sphere model assuming a Schulz–Zimm size distribution. The best data fit is found for Au nanoparticles with a size distribution centred around 12.7 nm in diameter and a FWHM of the polydispersity peak of 2.5 nm.

**Figure 6:**
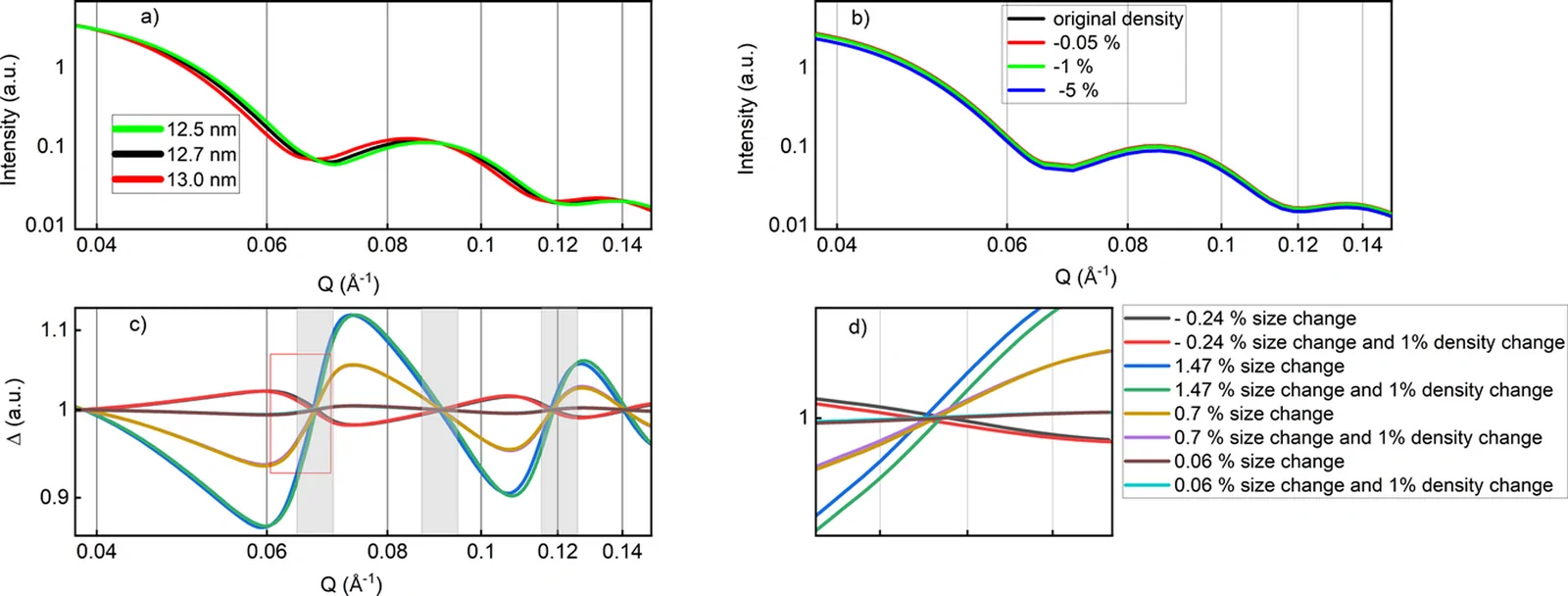
SAXS pattern simulations using *SasVIEW*. (*a*) Curves for three different Au nanoparticle diameters. (*b*) Curves for four different Au nanoparticle densities. (*c*) Signal division of the simulated SAXS curves for various particle size variations. A positive sign means the particles are larger than the reference and a negative sign means the particles are smaller. Particle size change is the factor that contributes the most to the curve shape. (*d*) A magnification of the region in panel (*c*) marked by a red rectangle, showing that particle density changes at constant volume only affect the intensity shift and move the curves vertically.

To determine and assess the accuracy of the size determination, SAXS patterns were simulated for hard spheres of varying diameters using *SasVIEW*. These simulations play a role in understanding the time-resolved measurements, where thermal expansion will occur due to the laser excitation. Scattering curves for three different sphere diameters are presented in Fig. 6 ▸(*a*), but particle size changes ranging from 0.05% up to 1.5% have been simulated. Fig. 6 ▸(*b*) shows simulated SAXS patterns with a constant particle size but varying density. Density reductions up to 5% from the nominal value were calculated. A change in density affects only the scattering intensity, and all the peaks and shapes stay the same in the broad *Q* region 0.04 < *Q* < 0.1 Å^−1^. When evaluating the experimental data, the small time-resolved changes in size, resulting in small changes in the positions of the minima in the SAXS intensity profile, can be enhanced by dividing the data into a class before laser interaction and a class after the laser interaction and expansion have occurred. The simulated data were used in a similar way to guide the experiment. To identify regions where the changes are most pronounced in reciprocal space, the ratio signals were extracted by dividing the simulated scattering curves.

The resulting simulated ratio signal profiles are shown in Fig. 6 ▸(*c*). They have complex periodic features with a clear overlap at specific scattering vector magnitudes around *Q* = 0.066 Å^−1^, *Q* = 0.091 Å^−1^ and *Q* = 0.118 Å^−1^. The signal is most pronounced at small *Q* magnitudes and gets lower for higher *Q* values. These regions are marked in grey. They serve as a marker of particle size variation and are targeted in TR-SAXS experiments to resolve size changes induced by laser excitation. Small density variations (< 1%) cannot be resolved in the ratio signal and only particle size changes generate oscillations outside the grey shaded regions. In contrast, large density changes (> 5%) at constant volume result in an overall intensity shift of the entire curve and a change in slope at *Q* < 0.05 Å^−1^, while the peak positions remain unchanged.

## 7. Ultrafast TR-SAXS

The time-resolved X-ray measurements were performed at an energy density of 5 mJ cm^−2^ at 400 nm. The low laser fluence was chosen to avoid laser-induced sample damage, which can cause melting of Au nanospheres. A uniform sample excitation causes material expansion, resulting in different SAXS patterns at different time delays. The laser energy is deposited into the electron system of the gold particles. There is a subsequent thermal equilibration between the lattice and the electron subsystem through a process called electron–phonon coupling. The equilibration time depends strongly on the electron temperature, and differing times have been reported for gold thin films. The work published by Chen *et al.* (2011 ▸) reports maximum integrated intensity changes at 50 ps in 150 nm thick Au(111) crystals after 100 fs using laser heating at 400 nm. In comparison, work from Pudell *et al.* (2018 ▸) shows slower Au lattice heating in thin films, with the maximum signal reached after 80 ps in the thin-film structure. Times shorter than 10 ps were required to implement an X-ray switch on FemtoMAX (Jarnac *et al.*, 2017 ▸). Electron–phonon coupling times in nanomaterials ranging from 3 ps to 4 ps for 15 nm gold nanoparticles (Link & El-Sayed, 2003 ▸) have been reported. Hashimoto *et al.* (2012 ▸) calculated lattice temperature following femtosecond laser excitation in Au nanoparticles suspended in water, with a maximum lattice temperature achieved at around 30 ps which decays slowly over the next few hundred picoseconds.

TR-SAXS data were acquired for about 30 min (15000 shots, which was three times longer than the static SAXS measurements) for each time point at several time delays, in order to track sample size changes after laser excitation. The longer exposure time improves the S/N ratio in the region marked in grey in Fig. 6 ▸(*c*) according to the simulations (range 0.04 < *Q* < 0.1 Å^−1^).

**Figure 7:**
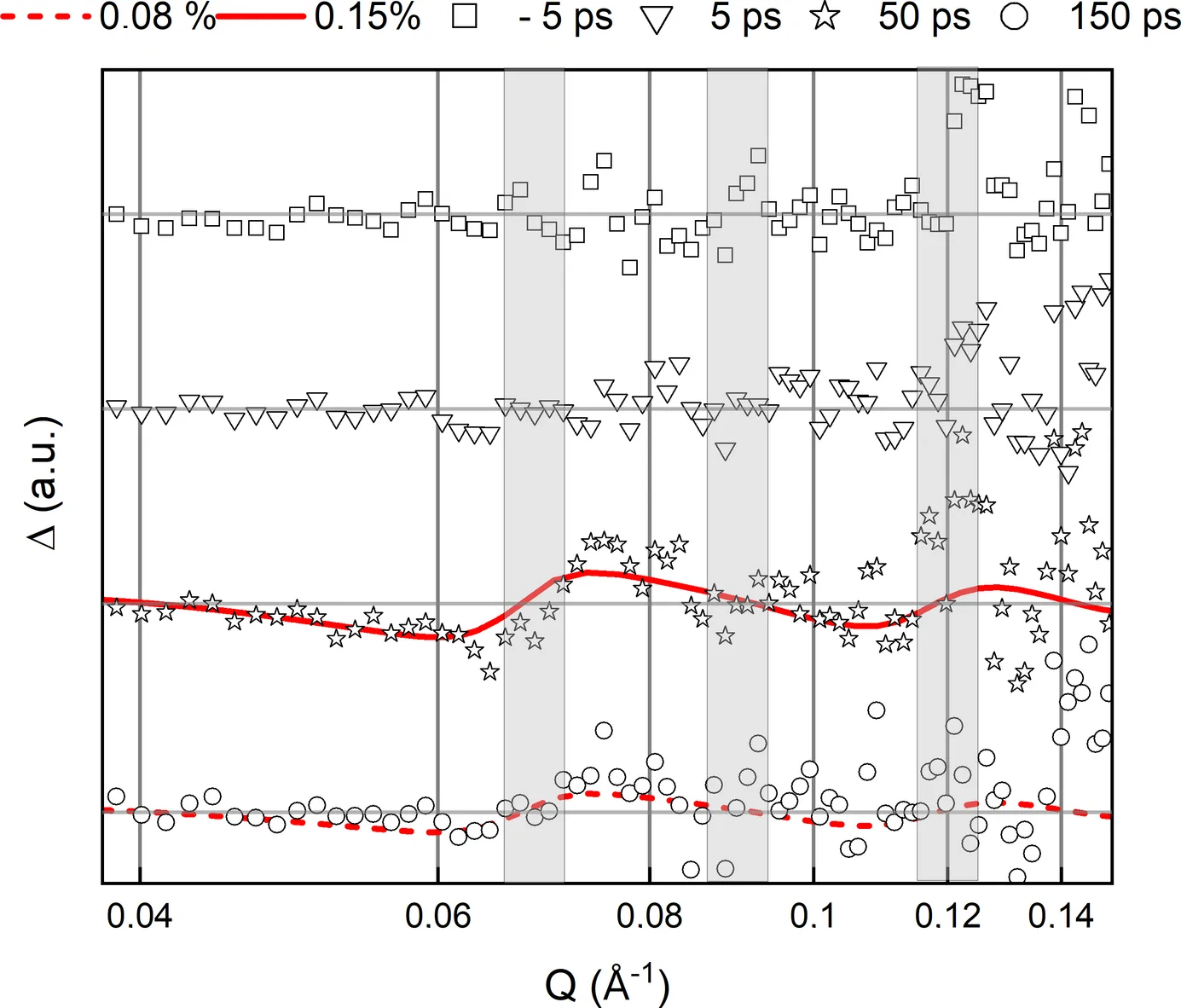
The ratio of two signals, laser on and laser off, as a function of *Q* at different time delays. Experimental data are shown as open symbols (squares −5 ps, triangles 5 ps, stars 50 ps and circles 150 ps), while the red solid and dashed lines represent calculations for average particle size expansions of 0.15% and 0.08%, respectively. The curves and datasets are vertically offset for clarity. Grey shaded regions mark the *Q* ranges where the calculated ratio signal remains unchanged (see Fig. 6 ▸). Outside these regions, the ratio signal increases at higher *Q* values and decreases at lower *Q* values. A hint of time-resolved signal is observed at 5 ps, indicating the possible expansion of the nanospheres shortly after laser excitation. Due to the small signal amplitude and poor signal-to-noise ratio, this time point was not considered in the quantitative analysis. The maximum thermal expansion of the nanospheres is observed at 50 ps after laser excitation and gradually decays at longer delay times as the temperature decreases. At 150 ps, the ratio signal remains detectable but is substantially weaker than at 50 ps and approaches the noise level.

We chose to investigate two longer times (50 ps and 100 ps) when we were confident that the maximum temperature had been reached, and a shorter time (5 ps) in order to look for non-thermal effects. A ratio signal from the laser-excited sample divided by the signal without laser excitation was used to extract time-resolved sample changes. The results are presented in Fig. 7 ▸. The first two grey shaded regions shown in the simulations in Fig. 6 ▸(*c*) can clearly be identified, while the next one is hidden in the noise. The overall shape of the signal division curve closely matches the simulated profiles shown in Fig. 6 ▸(*c*), confirming the validity of the modelling approach. The signal amplitude outside the grey shaded regions provides information about the actual particle size changes at specific time delays. Using *SasVIEW*, we could extract the temporal evolution of the particle dimensions at different time delays. The maximum change occurs approximately 50 ps after laser excitation. The ratio signal amplitude is relatively low, due to sample polydispersity and spatial inhomogeneities induced by the excitation pulse. Analysis of the TR-SAXS signal allowed us to estimate the average particle size variation within the probed volume at different time delays. A maximum relative size change of about 0.15% was observed at a probe delay time of 50 ps, followed by a gradual relaxation at later delays, which is consistent with earlier reports (Chen *et al.*, 2011 ▸; Plech *et al.*, 2003 ▸; Ligges *et al.*, 2009 ▸; White *et al.*, 2014 ▸; Brorson *et al.*, 1987 ▸; von Reppert *et al.*, 2016 ▸).

The agreement between our observations and the literature values supports the interpretation that the measured structural changes are governed by ultrafast energy transfer from the electrons to the Au lattice. The exact physical phenomena leading to a detailed understanding of how the deposited laser energy is transferred to the gold lattice do not fall within the scope of this paper and are therefore not discussed in detail.

Our findings demonstrate the capability of the FemtoMAX TR-SAXS setup to resolve time-dependent structural dynamics in non-uniform solids. This first demonstration experiment was performed on gold nanospheres as they are easily available, ultrafast laser dynamics in gold are well understood and gold is often use as a test sample for SAXS. The next step could be to study nanoparticles of a lighter element, such as aluminium or carbon, to show a broader applicability. At 2.5 keV, which is within the beamline energy range, the absorption contrast for Al is similar to that of Au at 12 keV.

## 8. Conclusions

In this work, we have demonstrated a significant step forward in time-resolved SAXS (TR-SAXS) measurements with picosecond temporal resolution on the FemtoMAX beamline. The scattering signal was recorded using a small-pixel detector from Andor, providing high *Q* resolution at the cost of a reduced signal-to-noise ratio, which was improved by implementing a single-photon counting algorithm. We have demonstrated the capabilities of the instrument using the Andor Balor detector by extracting the average Au particle size from static SAXS measurements and by capturing laser-induced thermal expansion in gold nanoparticles. Further instrument improvements could be achieved by employing more efficient X-ray detectors, such as a Dectris Pilatus or Dectris Eiger.

## Figures and Tables

**Table 1:** The present TR-SAXS setup configuration on the FemtoMAX beamline Provisions have been made to extend the sample-to-detector distance up to 6 m, but this high-*Q*-resolution setup has not been commissioned yet. The TR-SAXS setup can be equipped with the other 2D detectors available on the beamline, the Andor Zyla and Dectris Pilatus. The Andor Zyla, with a pixel size of 6.5 µm × 6.5 µm and a sensor area of 14 mm × 16 mm, offers ultra-high resolution at a cost of reduced *Q* magnitude. In contrast, the Dectris Pilatus, with a pixel size of 172 µm × 172 µm and a sensor area of 253 mm × 142 mm, offers larger *Q* magnitudes. Custom air and in-vacuum configurations can be prepared on request from the user.

Commissioned TR-SAXS setup configurations on the FemtoMAX beamline				
Detector name, Pixel size, No. of pixels	Sample-to-detector distance	*Q*_max_ (Å^−1^)	Calculated momentum resolution Δ*Q* (Å^−1^)	Sample excitation options
Andor Balor, 12 µm × 12 µm, 4128 (W) × 4104 (H)		At 4 keV		400 nm, 800 nm, 1200 nm, 2.1 THz
	0.5 m	0.14	5 × 10^−5^	
	3 m	0.02	9 × 10^−6^	
Andor Balor, 12 µm × 12 µm, 4128 (W) × 4104 (H)		At 12 keV		
	0.5 m	0.35	2 × 10^−4^	
	3 m	0.07	3 × 10^−5^	

**Table 2:** Estimation of the incoming X-ray flux compared with the detected signal on the Andor Balor detector The incoming X-ray flux per pulse at 11 keV on the FemtoMAX beamline is 720000 photons at the sample position.

Factors reducing incoming X-ray signal	X-ray transmission	Absolute number of photons
Transmission via capillary (40 µm)	89%	640000
Transmission via sample Au (0.3 µm)	95%	608000
Transmission via light shield Al (40 µm)	81%	492000
1/4 deposited on the detector	25%	123000
Estimated detector QE at 11 keV, assuming 30 µm Si thick sensor	20%	25000

**Table 3:** A summary of the data-processing workflow The initial treatment of the raw detector output is followed by theoretical simulations using the *SasVIEW* software to extract laser-induced signal modulations imprinted in the scattered X-ray patterns recorded with the Andor Balor detector.

Step	Function	Outcome
Lowering static image background	Subtracting a constant value (median value) from every pixel in each raw Balor image	Flat image with background around 0 value
Image thresholding	Finding pixels with intensities above the threshold in each background-subtracted image	A map of photon hits on the detector
Refinement of single photon hits	Defining single photon hits by finding areas of 3 × 3 or 5 × 5 pixels where the sum value exceeds a second threshold (1100)	Noise-free images and a map of photon hit coordinates for each frame
Summing noise-free single images	Summing single noise-free images for each time delay	A sum image containing multiple X-ray pulses is saved (Fig. 4)
Radial integration	Radial integration of the summed images.	Radial line outs representing *Q* versus intensity (Fig. 5)
Ratio signal extraction	Laser on/laser off	Ratio signals at different time delays (Fig. 7, black curve)
Experimental data fitting using *SasVIEW*	Scattering profile calculations for different Au particle diameters and densities	Radial line outs representing *Q* versus intensity [Figs. 6(*a*) and 6(*b*)]
Time-resolved data fitting using *SasVIEW* calculated scattering profiles	Finding the best fit for the ratio signal by calculating Au particle size changes at different time delays for calculated SAXS patterns using *SasVIEW*	Ratio signals at different time delays representing different average Au particle size changes a specific time after laser excitation (Fig. 7, red curve)
